# The association of status transitions from school to work with leisure-time physical activity on weekdays: a longitudinal analysis of data from the German Socio-Economic Panel

**DOI:** 10.1186/s12889-025-23739-4

**Published:** 2025-08-02

**Authors:** Jessica T. Bau, Simon Götz, Adrian Loerbroks, Claudia R. Pischke

**Affiliations:** 1 Institute of Medical Sociology, Unit Public Health, Centre for Health and Society, Medical Faculty, University Hospital Düsseldorf, Heinrich Heine University Düsseldorf, Moorenstr. 5, Düsseldorf, 40225 Germany; 2 Institute of Medical Sociology, Centre for Health and Society, Medical Faculty and University Hospital Düsseldorf, Heinrich Heine University Düsseldorf, Düsseldorf, Germany; 3 Institute of Occupational, Social and Environmental Medicine, Centre for Health and Society, Medical Faculty, University Hospital Düsseldorf, Heinrich Heine University Düsseldorf, Düsseldorf, Germany

**Keywords:** Physical activity, School-to-work, Transitions, Longitudinal analysis, Starting employment

## Abstract

**Background:**

Physical inactivity is common among young adults. Transitions from school to work can affect available resources for engaging in physical activity (PA). There is a lack of longitudinal data examining changes in PA following status transitions differentiated by occupational and academic career paths. The aim of this study was to analyze changes in PA of young adults across four status transitions from: (1) school to vocational education and training (VET), (2) school to university, (3) VET to workforce entry, and (4) university to workforce entry.

**Methods:**

A longitudinal analysis was conducted using data from the German Socio-Economic Panel (waves 2014–2020) for young adults aged 18–35. McNemar tests and logistic fixed-effects regressions were performed in four separate subsamples with paired data (time points before and after a transition).

**Results:**

In total, 364 individuals transitioned from school to VET (47.8% women, mean age before transition (BT): 18.9, SD = 1.2), 482 from school to university (53.7% women, mean age BT: 19.0, SD = 1.4), 790 from VET to workforce entry (46.6% women, mean age BT: 22.2, SD = 3.3), and 305 from university to workforce entry (54.4% women, mean age BT: 26.3, SD = 3.0). A significant reduction in PA was observed when individuals transitioned from 1) school to VET and 4) university to workforce.

**Conclusions:**

Young adults who choose an occupational career path already experienced a reduction in PA when starting VET, whereas this trend is temporally shifted for those choosing an academic career path, occurring only upon entry into the workforce. Tailored interventions (e.g., digital approaches that are flexible in terms of time and location) are needed to promote PA among the identified groups, considering available resources.

**Supplementary Information:**

The online version contains supplementary material available at 10.1186/s12889-025-23739-4.

## Background

Regular physical activity (PA) is crucial for maintaining health and preventing chronic non-communicable diseases [1], such as type 2 diabetes [2], certain cancers [3] or cardiovascular diseases [4], but also mental disorders, such as depression [5] or cognitive impairments, such as dementia [6].

Accordingly, the World Health Organization recommends that adults should engage in 150–300 min of moderate-intensity or 75–150 min of vigorous-intensity PA per week in combination with regular muscle-strengthening activities [7]. With increasing age, the percentage of those meeting these recommendations decreases. However, already in young adulthood (ages 18–29), approximately 40% of women and 30% of men in Germany do not engage in sufficient PA [8]. To develop appropriate interventions for PA promotion, it is important to understand when and why individuals experience a decline in PA and identify personal and environmental factors contributing to a reduction in PA [9, 10].

Young adulthood is a stage in life characterized by various critical life events, such as moving out of the parental home, starting post-secondary education or employment, getting married or becoming a parent. Life events like these can be understood as transitions from one status to another (e.g., student - employee, single - married) which may result in changes in behavior patterns [9, 11]. As a major life event, first entry into the workforce involves changes in daily routines and a shift in personal resources, such as availability of time and energy, which may negatively affect engagement in PA [12]. A full-time job takes up a substantial amount of time each day, especially when individuals have to work overtime or have to commute to their workplace. According to the Eurobarometer on Sport and Physical Activity, an annual public opinion survey conducted by the European Commission, a lack of time is the main reported barrier for PA among respondents from 27 EU Member States [13]. Additionally, decisions on how to use one’s scarce leisure time are influenced by a calculation of social payoffs as the outcomes of a person’s social interactions. For instance, someone may prioritize spending time with a partner or networking with colleagues after work instead of doing sports. Also, if starting a new job involves relocating, previous social resources that motivated sports participation, such as membership in a local sports club, may then no longer be available [12, 14]. Taking up employment may also be accompanied by increased prolonged sitting, which is associated with higher morbidity and mortality, particularly among individuals aged 21–65 years and those with a lower socioeconomic status [15].

Gropper et al. [9] conducted a scoping review on the impact of (education-, employment-, relationship- and family-, residence- and health-related) life events on PA. Focusing on employment-related events, most studies found a decrease in PA levels for young adults entering the labor market. However, some studies solely focused on the analysis of subpopulations (e.g., women [16–18] or university professors [19]), only used retrospective data which is prone to information bias [14, 20] or employed cross-sectional designs that are inherently unsuitable to gain insights into potential causality [17]. Using a longitudinal design, Paluch et al. [21] found that starting a first job led to a significant decrease of moderate to vigorous PA at the within-individual level by an average of around 20 min per day among a population of US-American young adults aged 21–35 years who were repeatedly surveyed over a 12-month study period. In addition, Van Houten et al. [12] found that the frequency of sport declines by around 16% when an individual starts working more than 32 h per week in a Dutch population of individuals aged 15–45 years.

Reviewing the scientific literature, it becomes evident that **‘**beginning to work**’** is often not clearly defined. To understand developments in PA during the transition from school to work, different patterns ought to be considered. After lower, intermediate and upper secondary school, young adults can directly enter the workforce; however, more commonly, they first undergo vocational education and training (VET) or engage in higher education, i.e. enter university. Those who undergo VET in Germany already have an employee status, often work full-time (including vocational education), albeit with lower remuneration. Those who enter university often engage in part-time work to finance their studies. With the successful completion of post-secondary education, a second and often final transition into full-time employment occurs.

A decline in PA is already found during the transition from secondary school to university. Longitudinal studies from several countries compared levels of PA in the last year of high school, i.e. upper secondary school, and the first year of university (U.S.: [22, 23], Belgium: [24, 25], Australia: [26], UK: [27]. For Germany, we only found one study investigating PA during this transition using cross-sectional data, asking university students whether PA differed between the last year in upper secondary school and the first year in university. In this study, 45% of participants reported being less active during the first year at university [28]. Young adults who do not enter university after graduating from secondary school frequently undergo VET [29]. Here, studies indicate that vocational apprentices are even less physically active than university students with differences increasing over the duration of training periods [30]. One contributing factor is that adolescents who pursue VET often come from families with a lower socioeconomic status, which is associated with lower levels of health-promoting behaviors [29, 31].

To date, no longitudinal analysis has been conducted in Germany that examines engagement in PA during transitions from secondary school to post-secondary education, such as VET or university, and subsequently into the workforce. Therefore, the aim of our study was to explore how PA engagement develops throughout these transitions in a representative sample of young adults in Germany.

## Methods

### Data

We drew on data from the Socio-Economic Panel (SOEP), a representative longitudinal study of private households in Germany, conducted yearly since 1984, which originally focuses on social change and the development of living conditions. The study is funded by the German Federal Ministry of Education and Research (German: Bundesministerium für Bildung und Forschung; BMBF) and based at the German Institute for Economic Research (German: Deutsches Institut für Wirtschaftsforschung; DIW Berlin). The data collection is conducted by the Institute for Applied Social Science (German: Institut für angewandte Sozialwissenschaften GmbH; infas), using a mixed-mode design, combining computer-assisted as well as paper-and-pencil interviews. All samples are drawn using multistage random sampling, clustered regionally. Respondents are selected via random-walk or by register sampling [32, 33]. The SOEP adheres to EU regulations. All participants gave informed consent prior to participation. For more information about the SOEP, please visit diw.de/en/soep [34].

### Study sample

**Fig. 1:**
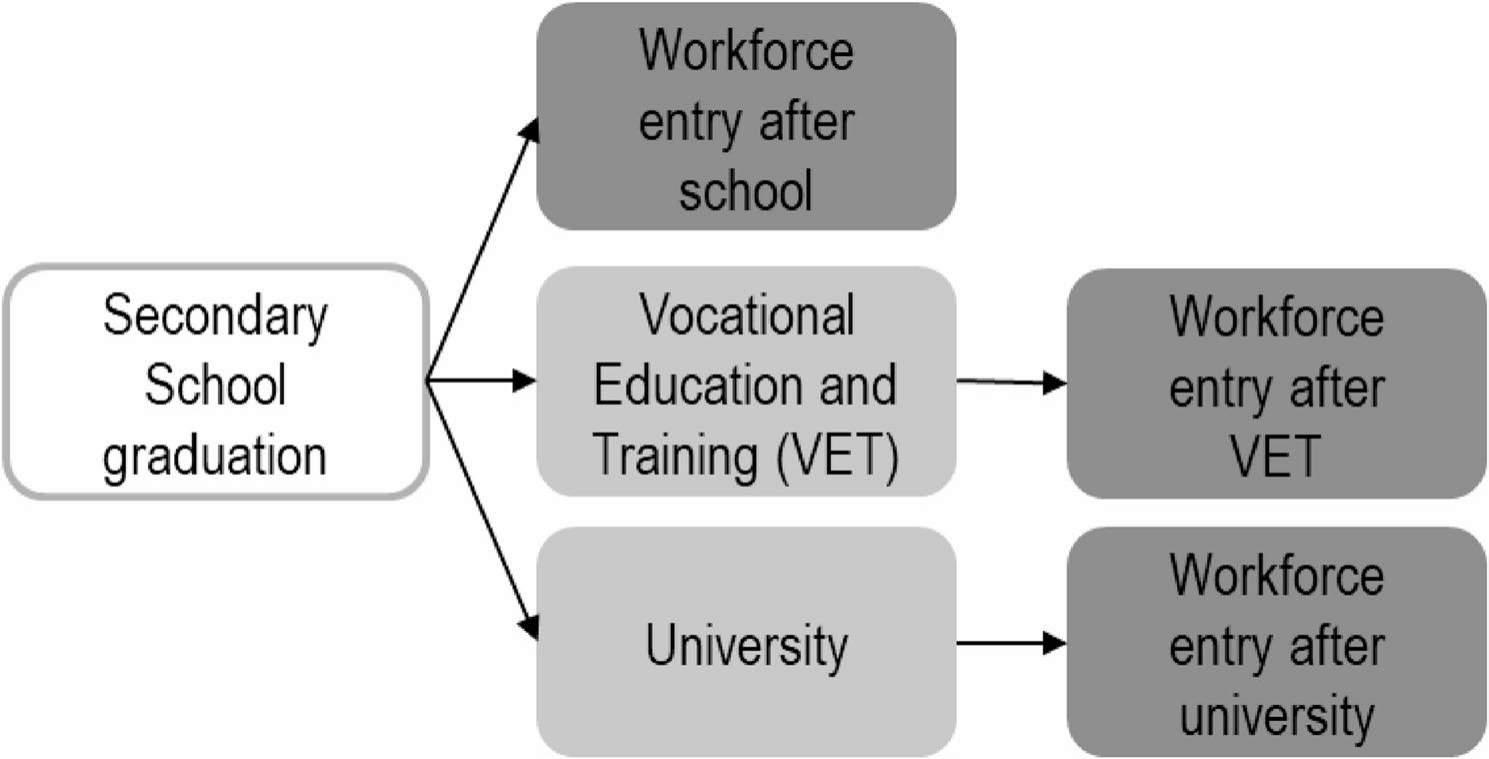
Pathways to employment in Germany

Panel waves from 2014 to 2020 were included in the analysis, because information on PA and educational status required were only collected since 2014 and the authors decided not to include the last available wave from 2021 to minimize the potential impact of the Covid-19 pandemic. The SOEP surveys individuals above the age of 18 years. To examine transitions in young adulthood, individuals above the age of 35 years were excluded from the analysis. Generally, respondents were included, if they had no missing values in the variables of interest. In numbers: A total of 55,130 unique individuals were interviewed in the SOEP across all samples between 2014 and 2020. After excluding individuals older than 35 years (exclusion of 33,211 individuals) and individuals with missing data on variables related to PA (exclusion of 100 individuals) or information on status transitions of interest under the specified conditions (see Additional file 1, exclusion of 2,710 individuals), the sample was reduced to 18,500 individuals. Most respondents were not interviewed across the entire period (due to dropouts, missing data, or later entry). Because this analysis focuses on status transitions (see Fig. 1), paired data were utilized, meaning two time points were considered (one before and one after the transition). This approach allows for the inclusion of individuals for whom only information on one of the transitions was available (e.g., only the first transition from secondary school to post-secondary education, but not into the workforce). However, as only the final year of secondary school is paired with the first year of post-secondary education, and the last year of post-secondary education is paired with the first year after workforce entry, the sample is ultimately reduced to 1,941 individuals with 3,882 observations. All other time points with observations (e.g., all years as an apprentice that are neither the first nor the last) were not included in the analysis. 16,559 individuals were excluded as they did not experience a relevant status transition during the observation period.

### Variables

The dependent variable *physical activity* was assessed using the question “What is a typical weekday like for you? How many hours per normal workday do you spend on the following activities?“. Besides the sub item of interest “Physical activities (sports, fitness, gymnastics)“, respondents were asked to provide information on time spent at their job, vocational apprenticeship or education (school/university), as well as housework, errands, and care work (children/elderly). The focus of this analysis was on leisure-time PA as opposed to occupational and transport PA, which were not surveyed sufficiently. The analysis focused on PA on weekdays, as the authors assumed a greater effect of status transitions on weekday routines and, beyond that, PA on weekends was only surveyed every two years. The number of hours of PA reported originally ranged from 0 to 24 hours per day. Records of more than 8 hours of PA per day were considered as invalid (even as a full-time competitive athlete) and were excluded from the analysis. Due to the rudimentary scaling (number of hours) used in the SOEP, the authors assumed that individuals who reported being physically active for 1 hour or more per day met the WHO recommendations for PA [7]. Consequently, *physical activity* was dichotomized: 0 for inactive and 1 for active (up to 8 hours of PA per day).

Figure 1 illustrates pathways to employment in young adulthood and possible status transitions in Germany. After lower, intermediate or upper secondary school, one possibility is to directly enter the workforce. However, school graduates are more likely to pursue VET or start studying at a university. In this case, workforce entry takes place after completion of post-secondary education.

As most young people complete secondary school to pursue further qualifications, we focused our analysis on the following transitions, creating four subsamples:

1. Secondary school to VET, including:
  Secondary school students in their final year before starting VET
  First-year vocational apprentices after secondary school graduation

2. Secondary school to university, including:
  Secondary school students in their final year before starting university
  First-year university students after secondary school graduation

3. VET to workforce, including:
  Vocational apprentices in their final year of training before entering the workforce
  First-year employees after VET completion

4. University to workforce, including:
  University students in their final year before entering the workforce
  First-year employees after university graduation

According to the groups listed in the subsamples, dichotomous variables (0/1) were created representing a respondents’ status. Conditions were set for maximum or minimum working hours, e.g., for job starters, who were expected to work at least 50% of the regular working hours to be considered employed. A detailed overview of the variables and the original items used in the SOEP as well as additional conditions set are provided in detail in Additional file 1.

Descriptive analysis provides information about socio-demographic characteristics, such as *age*,* gender* (0/1 = female), *migration background* (0/1 = individuals who immigrated to Germany themselves or with at least one parent with a migration background), and information about factors potentially influencing time resources: *partner in the household* (0/1 = yes*)*,* childcare* (0/1 = one or more hours of childcare on a typical weekday), *housework* (0/1 = one or more hours of housework on a typical weekday) and *caring for relatives* (0/1 = one or more hours of caring for relatives on a typical weekday). The analysis is complemented by variables describing health-related characteristics: *BMI (Body Mass Index)*, currently *smoking* (cigarettes, e-cigarettes, pipes or cigars; 0/1 = yes) and *drinking* (0/1 = drinking on two to three days a week or more).

### Statistical analysis

All analyses were conducted using Stata 16.1 (StataCorp LLC, College Station, Texas, USA). Descriptive statistics provide an overview of the distribution of sociodemographic, resource- and health-related characteristics (including the dependent variable PA) across all waves, separated by subsamples using percentages or means and standard deviations (SD). Since the health-related covariates were only surveyed biennially or less frequently, they were not included in further analyses.

McNemar tests *(mcc)* were used to examine whether there is a significant change in PA before and after the transitions, comparing the number of individuals who change from active to inactive and from inactive to active. Because panel data are nested within persons (same subjects measured repeatedly at different time points), logistic fixed-effects regressions *(xtlogit*,* fe or)* were used to control for time-invariant unobserved heterogeneity. In other words, participants served as their own controls and were not compared to others by using within-individual variance only. By this approach, we accounted for level differences in the dependent variable and controlled for unit-based serial dependence [35]. Fixed-effects models have the advantage that they do not only account for known time-invariant covariates, such as gender and migration background, but also unknown covariates, such as intelligence or influences of primary socialization influencing health literacy. Analyses were calculated separately for each subsample. Results are presented as Odds Ratios (OR). Odds are defined as the expected frequency of an event occurring divided by the expected frequency of it not occurring [36]. The OR compares the odds between different groups– in this case, the odds of being physically active between young adults before and after a transition. Based on our theoretical considerations, we initially intended to include resource-related covariates (i.e. *partner in the household*,* childcare*,* caring for relatives and housework*) in the logistic fixed-effects regressions. For each of the four subsamples, we examined how many individuals experienced changes in these covariates during a transition (e.g., having no partner before starting VET, but having one afterward) and used McNemar tests to examine whether differences were statistically significant. Due to the small number of individuals who experienced a change in partnership, childcare or caring for relatives, including these variables in the regression model either resulted in extremely large standard errors and wide confidence intervals, or led to their automatic exclusion due to insufficient within-person variation. While more changes in housework were observed (both taking on and giving up housework), the inclusion of this variable in the model also did not yield statistically significant results. Additionally, the effects of the main explanatory variables remained robust across all specifications. We therefore decided to exclude the covariates from the final analyses.

## Results

Table 1 provides an overview of the characteristics of the different subsamples.

**Table 1:** Individual characteristics of the subsample used from the German Socio-Economic panel, waves 2014–2020

	Subsample 1		Subsample 2		Subsample 3		Subsample 4		
	Last-year school students^a^	First-year apprentices	Last-year school students^b^	First-year university students	Last-year apprentices	Job starters after VET	Last-year university students	Job starters after university	
n (individuals)	364	364	482	482	790	790	305	305	
Socio-demographic characteristics									
Age; mean (SD); (min, max)	18.90 (1.24); (18, 26)	20.27 (1.33); (19, 28)	18.98 (1.43); (18, 30)	20.33 (1.48); (19, 31)	22.18 (3.33); (18, 34)	23.38 (3.33); (19,35)	26.34 (3.01); (19, 34)	27.54 (3.01); (20, 35)	
Female (%)	47.80	47.80	53.73	53.73	46.58	46.58	54.43	54.43	
Migration background (%)	27.20	27.20	29.25	29.25	33.54	33.54	32.79	32.79	
Resources-related characteristics									
Working (%)^c^	12.36	100.00	10.58	18.46	100.00	100.00	36.72	100.00	
Weekly working hours; mean (SD)	11.46 (10.51)	38.56 (4.06)	9.78 (9.74)	9.51 (4.08)	38.84 (3.21)	38.32 (4.34)	11.58 (4.07)	35.20 (7.75)	
Commuting > = 20km^d^	4.00	32.68	17.24	8.33	26.32	30.75	20.69	28.24	
Partn. i. household (%)	1.65	4.40	2.07	4.56	18.23	23.16	33.44	44.92	
Childcare (%)^e^	9.07	4.95	8.09	4.56	8.35	7.97	9.51	10.82	
Housework (%)^f^	60.17	65.84	64.45	77.29	64.05	70.92	88.04	84.87	
Caring for relatives (%)^g^	1.10	1.38	2.90	3.73	2.79	2.41	2.62	0.66	
Physical activity									
Physically active (%) ^h^	69.23	60.16	75.31	72.41	53.16	55.32	69.51	63.28	
Further health-related characteristics ^i^									
BMI (%)^j^									
* Underweight*	8.25	7.53	9.35	7.42	3.20	2.62	3.77	1.90	
* Normal weight*	69.07	70.43	76.42	69.14	62.07	58.79	66.67	62.66	
* Overweight*	18.04	18.28	10.57	17.58	22.17	24.15	25.79	29.11	
* Obesity*	4.64	3.76	3.66	5.86	12.56	14.44	3.77	6.33	
Smoking %^k^	18.56	25.52	9.02	7.28	36.50	36.11	17.72	18.01	
Alcohol Drinking %^l^	18.52	19.38	16.42	13.45	15.32	21.88	15.38	26.27	

^a^School students in their last year, who become first-year apprentices in VET in the next year

^b^School students in their last year, who become university students in the next year

^c^Working = At least 1 working hour per week

^d^Information on commuting was only available for the years 2015, 2017, 2019; sample sizes n (years) respectively: school students before VET = 25; first year vocational apprentices = 153; school students before university: 29; first-year university students = 48; last-year vocational apprentices = 323; job starters after VET = 348; last-year university students = 58; job starters after university = 131

^e^Childcare = One or more hours of childcare (own children, siblings) on a regular weekday

^f^Caring for relatives = One or more hours of caring for relatives on a typical weekday

^g^Housework = One or more hours of housework on a typical weekday

^h^Physically active = One or more hours of sports, fitness or gymnastics on a regular weekday

^i^Information on (1) BMI and (2) smoking was only available for the years 2014, 2016, 2018, 2020; (3) information on drinking was available for the years 2016 & 2020; sample sizes n (years), respectively: last year school students before VET = 194/194/67; first-year vocational apprentices = 186/192/130; last-year school students before studies = 246/250/67; first-year university students = 256/261/171; last-year vocational apprentices = 406/400/124; job starters after VET = 380/396/256; last-year university students = 159/158/52; job starters after university = 158/161/118

^j^BMI = Body Mass Index; calculated by dividing a person’s weight (in kg) by the square of their height (in m). The World Health Organization classifies the following categories: Underweight: BMI < 18.5; Normal weight: 18.5–<25, Overweight: BMI ≥ 25–<30; Obesity: BMI ≥ 30 (Class 1: BMI 30–<35; Class 2: BMI 35–<40; Class 3: BMI ≥ 40) [37]

^k^Smoking = Currently smoking cigarettes, e-cigarettes, pipes or cigars

^l^Drinking = Drinking alcohol two to three days a week or more

The total sample consisted of participants between the ages of 18 and 35 years. In accordance with the timing of events, the respective reference groups in the subsamples were younger than the comparison groups after transition. Among school students, the vast majority (88%) attended upper secondary school or comprehensive school, 6% attended intermediate secondary school, 4% attended lower secondary school, and 2% attended another type of school (not shown in Table 1).

According to the assumption that leisure-time PA is related to available time resources, we examined whether and to what extent members of the respective subsamples were working. As much as 100% of job starters and 100% of vocational apprentices reported working, each between an average of 35–39 h. 12.4% of school students in their final year before starting VET and 10.6% of those entering university in the following year were employed, working an average of 11.5 and 9.8 h, respectively. Furthermore, 18.5% of first-year and 36.7% of last-year university students reported working an average of 9.5 and 11.6 h weekly. Between 4% (last-year school students before starting VET) and 30.8% (job starters after completing VET) reported commuting more than 20 km to work.

Regarding further resources-related characteristics, the proportion of those living with a partner in the same household increased with age and the completion of post-secondary education. The proportion of those who reported providing one or more hours of childcare on a regular workday varied between 4.6% (first-year university students) and 10.8% (job starters after university graduation). Among school students, childcare probably involved caring for younger siblings. Housework was done by 60.2% of last-year school students before starting VET (lowest proportion) and 88.0% of last-year university students (highest proportion). Caring for relatives played a minor role in the availability of resources.

**Fig. 2:**
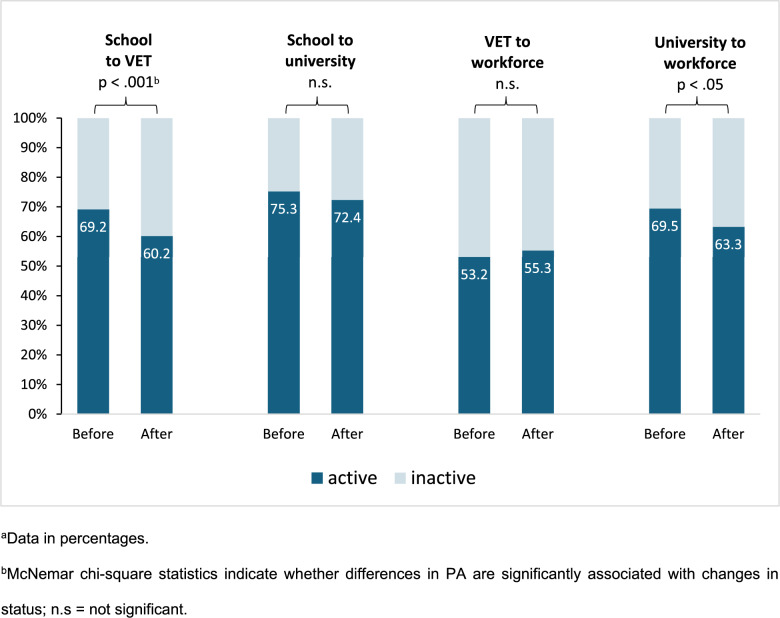
Physical activity before and after transitions: McNemar’s test^a^

Table 1 (and Fig. 2) also shows the proportion of individuals who reported being physically active. With 75.3% of individuals being active, last-year school students prior to entering university are the most active, followed by first-year university students at 72.4%. Apprentices before and after completing VET and entering the workforce are the least active, with 53.2% and 55.3%, respectively.

The proportion of individuals classified as obese according to BMI is highest among last-year vocational apprentices (12.6%) and those entering employment thereafter (14.4%). Additionally, these groups also show the highest prevalence of smoking, with 36.5% and 36.1%, respectively. In contrast, only 17.7% of last-year university students smoked, but 26.3% first-year job starters after university graduation reported drinking alcohol on two or three days a week or more. It should be noted that the data on commuting, BMI, smoking, and alcohol drinking were not collected annually and refer to smaller subsamples due to missing values (see sample sizes in the footnote of Table 1).

Figure 2 compares the proportion of physically active individuals before and after transitions from school to work.

We found a significant association for the transition from school to VET (*p* = 0.0007). Of the 364 individuals in subsample 1, 64 (17.6%) experienced a change in PA from active to inactive, and 31 (8.5%) from inactive to active with the start of VET. 188 (51.7%) were active and 81 (22.3%) were inactive at both time points (not shown in Fig. 2). Overall, 69.2% of school students in their final year were active, but only 60.2% of first-year vocational apprentices. We found no significant association between transitions from school to university (*p* = 0.2159) and VET to workforce (*p* = 0.2794), but for the transition from university to workforce (*p* = 0.0370). Of the 305 individuals in subsample 4, 51 (16.7%) experienced a chance in PA from active to inactive, and 32 (10.5%) from inactive to active when entering the workforce. 161 (52.8%) were active and 61 (20.0%) were inactive at both time points (not shown in Fig. 2). Overall, 69.5% of university students in their last year reported being active, but only 63.3% of job starters after university graduation.

To control for known and unknown time-invariant covariates, we calculated four fixed-effects regression models (see Table 2).

**Table 2:** Main effects of status transitions on physical activity. Results of logistic fixed-effects regression

	Physical activity				
	OR	Std. Err.	*P*-value	95% CI	*N*(obs)
Starting VET	0.484	0.106	0.001	0.315–0.744	190
Starting university	0.803	0.143	0.217	0.567–1.138	256
Entering workforce after VET	1.148	0.146	0.280	0.894–1.474	494
Entering workforce after university	0.627	0.142	0.039	0.403–0.976	166

We found that young people who started VET had reduced odds (OR = 0.484, *p* = 0.001) for being physically active compared to their final year of school. For school students entering university, we found no significant effect on PA. We also found no association between entering the workforce after completing VET and the odds of being physically active. However, university graduates entering the workforce had lower odds of being physically active in comparison to themselves in their last year of studies (OR = 0.627, *p* = 0.039).

Due to low variation in other resource-related characteristics within individuals (e.g., constant partner status across both years), the estimators for additional initially intended time-variant covariates are not shown. In adjusted models, we found no significant effects for partner, children, housework and caregiving. Some estimators could not be calculated at all or were unreliable, that is, showing very high standard errors and very wide confidence intervals.

As previous studies found differences in the response to life events between men and women, moderated by different coping strategies for stress [12, 21], we conducted additional analyses including interaction effects with gender, but found no significant effects in all subsamples.

## Discussion

This study aimed to investigate how status transitions from school to work affect PA among young adults in Germany.

### School to post-secondary education

We found a significant reduction in PA for school students who became vocational apprentices, but not for university students. This may be because starting VET leads to more comprehensive changes in an individual’s daily routine than starting higher education (i.e., university). While attending school or university generally allows for more time for engaging in leisure-time PA, VET is often more time-consuming and thereby restrictive. Even though some first-year students also engaged in employment alongside their studies, they reported spending, on average, only about a quarter of their time working compared to first-year vocational apprentices, who were often employed full-time. Additionally, vocational apprentices in our study were more likely to travel longer distances to their workplaces (one in three reported commuting more than 20 km to work), which consumes additional time resources. In general, we have no information whether participants chose active (e.g., walking, cycling) or passive (e.g., car, train) transport modes when commuting, however, with a greater distance, it is more likely that passive forms were chosen. While it can be assumed that university students spend a large part of their time sitting, doing desk work, we have no information about whether or to what extent the vocational apprentices in our study were physically active during their professional activities. But even if they were physically active at work (e.g., in crafts), recent studies suggest that occupational PA can have detrimental health consequences (PA paradox) [38]. In an umbrella review, researchers found that high occupational PA was associated with all-cause mortality in men, mental ill health, bad sleep quality, and osteoarthritis [39].

### Post-secondary education to workforce

Those who were previously engaged in VET did not experience a significant change in PA when entering the workforce. Starting a job after VET completion does not seem to bring changes in daily routines and, thus, no changes in PA. It is quite conceivable that some apprentices were retained by their employer after completing their training, meaning they continued to perform the same job. In contrast, we found a significant effect of entering the workforce after graduating from university on PA. In comparison to young adults completing VET, the reduction in PA for those pursuing a university degree appears to be temporally shifted, occurring after post-secondary education rather than after secondary education. While the daily routine of a university student seems to resemble that of a school student, fixed and long working hours might lead to less time for PA. Furthermore, it is conceivable that individuals entering the workforce after university graduation often relocate and/or become integrated in new social environments. As a result of these changes, physical activity– previously pursued in the company of peers in a similar stage of life– may become less central. Instead, after-work socializing may increasingly involve different activities, such as going to restaurants or bars with colleagues, rather than engaging in sports or visiting fitness facilities.

In general, young adults pursuing an occupational career path appear particularly prone to adopting behaviors detrimental to health. In our study, they were not only the least physically active but also reported the highest smoking rates and BMI scores. An occupational career is often concomitant with a lower socio-economic status. A scoping review by Matos Fialho and colleagues showed that individuals with a lower SES (often coming from low socio-economic status families) were more likely to engage in substance use (smoking, alcohol, cannabis), poor dietary behavior (fruits and vegetable consumption), and had higher body weight [31].

### Limitations and strengths

A major limitation of our study is that the SOEP measures PA solely in terms of hours per day, which led us to decide to dichotomize the dependent variable. Therefore, when interpreting the results, it should be considered that even those who reported 0 h of leisure-time PA per day may still meet the WHO recommendations. Calculated per day, a person would need to be physically active for about 20 min to meet the minimum of 150 min of light to moderate PA per week. We also considered whether individuals might reduce PA on weekdays but compensate for it on weekends. However, as weekend PA was only assessed every two years (2015, 2017, 2019), we were unable to conduct the same analyses. Using the available data, we compared the proportions of individuals who were physically active on weekends before and after the transitions. Except for first-year university students– already the most active group– weekend activity declined post-transition. Overall, individuals were less active on weekends compared to weekdays. Future studies should use objective measurements (e.g., accelerometers) instead of subjective reports, examine PA throughout the entire week and consider PA intensity, i.e., differentiate between light to moderate and moderate to vigorous PA. Another limitation concerning the available information on PA is that only leisure-time PA is surveyed. Consequently, we were unable to investigate whether and to what extent individuals are physically active during working hours or if they engaged in active forms of commuting.

It should also be noted that we focused on the two most common pathways from school to work in Germany. We initially considered analyzing another transition, secondary school graduates entering the workforce directly, but decided to exclude this group from the analysis for both practical and theoretical reasons. Case numbers were very limited (10–20 individuals per year), and the group would have been highly heterogeneous. It would have included unskilled labor market entrants, individuals completing a voluntary social year, as well as those who had previously completed VET and later obtained an upper secondary school certificate before re-entering the workforce. Further research could explore additional transitions, such as the development of PA in individuals who first complete VET and then attend university.

Additionally, further studies should also consider potentially relevant time-variant covariates in fixed-effects regressions to examine the influence of resource- and health-related variables. In our analysis, reliable coefficients for time-variant covariates could not be calculated because information was either not collected annually or there was insufficient variance within the subsamples.

As we only examined the first year after a status transition, we cannot make any statements about whether a “recovery effect” occurs in the following years. We do not know if individuals become physically active again after a period of adjustment and adaption. Further research could investigate how PA develops in the years following a status transition. It would also be interesting to explore whether there are differences between various university courses or VET programs, i.e., in which industries or sectors young people are at particular risk of becoming physically inactive.

In order to avoid potential misinterpretation, particularly an overestimation of effect sizes due to the high prevalence of the outcome (physical activity among young adults), we would like to emphasize that the ORs reported in our analysis do not correspond to relative risks (RRs), understood as ratios of probabilities [40]. We opted to report ORs to allow for comparability with prior studies [12, 14, 22, 27, 30], but the results of a fixed-effects modified Poisson regression *(xtpoisson*,* fe vce(robust) irr)* estimating relative risks can be found in Additional file 2. As expected, the results are similar across analysis approaches.

We would like to note that our sample was drawn from a large, representative, population-based survey. A comparison of our subsamples with the general population shows that the distributions of gender [41], migration background [42], and geographic regions [43] are largely comparable to those of the general population of young adults in Germany. However, graduates of upper secondary schools are overrepresented in subsample 1, as only individuals aged 18 years and older were surveyed, and lower and intermediate secondary school qualifications are typically completed at an earlier age. As only individuals who experienced a specific transition and a change in physical activity could be included in the logistic fixed-effects regression, it is possible that further statistically significant associations might be observed with larger samples.

Finally, we would like to note that, although we used data from 2014 to 2020, we were not able to take secular trends in PA into account, as our analyses focused exclusively on changes within a narrow time frame (i.e., data from individuals observed in two consecutive years). According to the Federal Statistical Office, the average time spent on sports has increased by approximately five minutes between 2012 and 2022 [44]. However, as PA in our study was measured in hours, it is likely to be less sensitive to subtle trends.

Despite the mentioned limitations, this study is, to the best of our knowledge, the first longitudinal study that investigates the development of PA from school to workforce, divided into separate status transitions. The logistic fixed-effects regression allowed for the control of all time-constant factors and the within-individual comparison over several years.

## Conclusions

Status transitions may influence an individual’s available resources for engaging in PA. In our study, we identified two groups of young adults who experienced a reduction in PA during the transition from school to work: Secondary school graduates entering VET and university graduates entering the workforce. The timing of the decline in PA varies depending on the educational path chosen, occurring earlier among those pursuing an occupational career. We assume that life circumstances change after a transition. There is probably less leisure time available due to long working hours and commuting, as well as due to time spent establishing new social ties. Tailored interventions (e.g., digital approaches that allow for flexibility in engagement in terms of time and location) are needed to take these changes into account and promote PA among the identified groups.

## Supplementary Information

Additional file 1. Status definitions and set conditions.

Additional file 2. Main effects of status transitions on physical activity. Results of fixed-effects modified Poisson regression estimating relative risks.

## Data Availability

The data from the Socio-Economic Panel are accessible for scientific purposes to all individuals working at research institutions. The data can be requested from the German Institute for Economic Research (German: Deutsche Institut für Wirtschaftsforschung; DIW Berlin).

## Abbreviations

PA: physical activity
VET: vocational education and training
BT: before transition
SD: standard deviation
