# Supplementary material for: The association of status transitions from school to work with leisure-time physical activity on weekdays: a longitudinal analysis of data from the German Socio-Economic Panel
Source: BMC Public Health. 2025 Aug 2;25:2624. doi: 10.1186/s12889-025-23739-4 (PMC12317488; doi:10.1186/s12889-025-23739-4)
Supplement: Supplementary file 1 — Additional file 1. Status definitions and set conditions. [file 12889_2025_23739_MOESM1_ESM.docx]

Additional file 1

**Status definitions and set conditions**

**Table S1: Status definitions and set conditions. Original items used in the socio-economic panel (SOEP).**

| **Independent variables** | **Items** | **Additional conditions** |
| --- | --- | --- |
| *Last-year school student* | - If you are currently in education or training: What type of education or training are you pursuing? -> Secondary school (lower, intermediate, upper) | - Currently enrolled in lower, intermediate or upper secondary school - Not a school student in the next year - Starting VET or entering university in the next year or the year after next (to account for potential delays, such as waiting for a spot) - Not a vocational apprentice (occurred in cases where individuals completed intermediate or upper secondary school during an apprenticeship, which was started after completing lower or intermediate secondary school) - No limitation on weekly working hours alongside school, as this is not regulated in Germany |
| *First- and last-year vocational apprentice* | - Are you currently employed? Which one of the following applies best to your status? -> Completing in-service training / apprentice-   ship / in-service retraining | - Currently employed as an apprentice - Weekly working >0 hours (thus, marginally employed or unemployed individuals are excluded, as well as those in purely school-based training, since we assumed no significant differences in comparison to school)   First-year:   - Not been in VET in the last year - Been a school student in the last or second-to-last year (to account for potential delays, such as waiting for a spot)   Last-year:   - No longer in any training or education in the next or the following year (i.e., school, VET, university) |
| *First- and last-year university student* | - Are you currently in education or training? What type of education or training are you pursuing?   -> University | - Currently enrolled at a university - Weekly working <=20 hours (students working more than 20 hours are counted as employees and are required to pay full social security contributions in Germany; during semester breaks, students are allowed to work more hours according to law, but we cannot verify whether an individual is currently in a semester or not)   First-year:   - Not been a university student in the last year - Been a school student in the last or second-to-last year (to account for potential delays, such as waiting for a spot)   Last-year:   - No longer in any training or education in the next or the following year (i.e., school, VET, university) |
| *Job starter after VET completion* | - Are you currently employed? Which of the following applies best to your status?   -> Employed full-time  -> Employed part-time | - Been an apprentice in the last or second-to-last year (to account for potential delays, such as searching for a job) - Weekly working >=17 hours (among vocational professions, we found the shortest regular full-time working hours in the metal and electrical industry, with 35 hours; we have decided not to consider someone who works less than 50% (rounded down: 17 hours) as a job starter) |
| *Job starter after university graduation* | - Are you currently employed? Which of the following applies best to your status?   -> Employed full-time  -> Employed part-time | - Been a university student in the last or second-to-last year (to account for potential delays, such as searching for a job) - Weekly working >=12 hours (among job starters after graduation, we found the shortest regular full-time working hours among teachers, with 25 to 28 hours; we have decided not to consider someone who works less than 50% (rounded down: 12 hours) as a job starter) |
