# Supplementary material for: The association of status transitions from school to work with leisure-time physical activity on weekdays: a longitudinal analysis of data from the German Socio-Economic Panel
Source: BMC Public Health. 2025 Aug 2;25:2624. doi: 10.1186/s12889-025-23739-4 (PMC12317488; doi:10.1186/s12889-025-23739-4)
Supplement: Supplementary file 2 — Additional file 2. Main effects of status transitions on physical activity. Results of fixed-effects modified Poisson regression estimating relative risks. [file 12889_2025_23739_MOESM2_ESM.docx]

Additional file 2

**Main effects of status transitions on physical activity. Results of fixed-effects modified Poisson regression estimating relative risks.**

**Table S2: Main effects of status transitions on physical activity. Results of fixed-effects modified Poisson regression estimating relative risks.**

|  | **Physical activity** | | | | |
| --- | --- | --- | --- | --- | --- |
|  | **RR** | **Std. Err.** | **P-value** | **95% CI** | **N(obs)** |
| **Starting VET** | 0.869 | 0.036 | .001 | 0.801–0.943 | 566 |
| **Starting university** | 0.961 | 0.031 | .216 | 0.903–1.023 | 840 |
| **Entering workforce after VET** | 1.040 | 0.038 | .279 | 0.968–1.118 | 552 |
| **Entering workforce after university** | 0.910 | 0.041 | .037 | 0.833–0.994 | 488 |
